# Clinical-Pathological Features and Outcome of Atypical Anti-glomerular Basement Membrane Disease in a Large Single Cohort

**DOI:** 10.3389/fimmu.2020.02035

**Published:** 2020-09-03

**Authors:** Cong-rong Shen, Xiao-yu Jia, Zhao Cui, Xiao-juan Yu, Ming-hui Zhao

**Affiliations:** ^1^Renal Division, Peking University First Hospital, Beijing, China; ^2^Institute of Nephrology, Peking University, Beijing, China; ^3^Key Laboratory of Renal Disease, Ministry of Health of China, Beijing, China; ^4^Key Laboratory of CKD Prevention and Treatment, Ministry of Education of China, Beijing, China; ^5^Research Units of Diagnosis and Treatment of Immune-Mediated Kidney Diseases, Chinese Academy of Medical Sciences, Beijing, China; ^6^Peking-Tsinghua Center for Life Sciences, Beijing, China

**Keywords:** anti-GBM disease, crescentic glomerulonephritis, rapidly progressive glomerulonephritis, renal outcome, renal pathology

## Abstract

**Background:** Atypical cases of anti-glomerular basement membrane (GBM) disease had absent circulating antibodies but linear IgG deposits along GBM in the kidneys. Herein, we reported the clinical-pathological features and outcome of these rare cases.

**Methods:** Linear IgG deposit along GBM were examined by immunofluorescence on renal specimens, with exclusion of diabetic kidney disease. Circulating anti-GBM antibodies were tested by commercial ELISA assay. Clinical, pathological and follow-up data were retrospectively analyzed.

**Results:** From 2013 to 2018, a total of 60 patients were diagnosed as atypical anti-GBM disease. They had a male predominance, with an average age of 51.7 ± 15.6 years. Three (5.0%) patients had alveolar hemorrhage. Forty five percent of them presented with acute kidney disease. All patients had linear IgG deposit along GBM, some in addition on tubular basement membrane and/or Bowmans' capsules. C3 deposition was found in 65.0% of the patients. 41.7% (25/60) of the patients showed crescent formation and the percentage of crescent was (34.7 ± 23.5)% in those patients. They had higher prevalence of hematuria and C3 deposit, higher levels of serum creatinine, worse renal and patient survival than those without crescent (*P* < 0.05). During the follow-up of 35.7 ± 21.4 months, 14 (23.3%) patients progressed to ESRD. The serum creatinine on diagnosis [per 200 μmol/L increase, HR (95% CI): 2.663 (1.372, 5.172), *P* = 0.004], serum C3 [per 0.1 g/L increase, HR (95% CI): 0.689(0.483, 0.984), *P* = 0.040] and the intensity of kidney C3 staining [per 1+ increase, HR (95% CI): 2.770 (1.115, 6.877), *P* = 0.028] were independent predictive factors for kidney outcome. Nine (15.0%) patients died of all causes.

**Conclusions:** Atypical anti-GBM disease manifested milder kidney injury and scarce pulmonary hemorrhage compared to the classical cases. Though heterogeneous, a substantial number of the patients had complement activation and crescent formation. Patients having crescents presented with more severe clinical course and worse outcomes. The poor kidney and patient prognosis emphasize prompt interventions from physicians. The immunosuppressive intervention was not associated with kidney or patient outcome. Further studies are needed to address the optimal therapeutic regimen.

## Introduction

Anti-glomerular basement membrane (GBM) disease is a rare *in situ* immune-complex vessel vasculitis that involves glomerular capillaries or pulmonary capillaries, or both (eponymously termed as Goodpasture syndrome) (1, 2). It is considered to be a prototypical autoimmune disease characterized by the burst of antibodies against the non-collagen domain one of α3 chain of type IV collagen [α3(IV)NC1] located in both GBM and alveolar basement membrane (3). The disease is documented as the most severe glomerulonephritis due to the rapidly progressive renal impairments with large amount of crescent in glomeruli and ~40~60% concurrence of lung hemorrhage including lethal massive hemoptysis (4). To improve kidney and patient outcomes, the combination regimen of plasmapheresis, steroids, and cyclophosphamide is recommended to start up immediately on diagnosis (5).

At present, the diagnosis of anti-GBM disease depends on the detection of circulating anti-GBM antibodies and/or linear IgG deposition along GBM on kidney biopsy (6). Clinical routine assay to detect circulating antibodies is enzyme-linked immunosorbent assay which utilizes recombinant human α3(IV)NC1 or purified bovine GBM as solid-phase antigen (7). The positive result is necessary for an early diagnosis and quick start of intensive treatments including plasma exchange and immunosuppressive therapy. However, in decades, atypical presentations of anti-GBM disease have been reported in case reports and case series (8–19), in which the circulating anti-GBM antibodies were often undetectable by commercial ELISA and the diagnosis was based on the linear deposit of immunoglobulins along GBM on renal specimens. The atypical condition brought challenges to the diagnosis and treatment of this aggressive disease. Whether these atypical cases are a homogeneous subtype of anti-GBM disease or a group of heterogeneous conditions is still not clear, nor are the causes and roles of the deposited antibodies in disease development. Therefore, it is of importance to explore their clinical and pathological characteristics and especially their outcomes from a large cohort.

In the present study, data from 60 consecutive “atypical” patients diagnosed from 2013 to 2018 were retrospectively screened, who presented with substantial linear deposits of IgG along GBM on immunofluorescence and without detectable circulating anti-GBM antibodies. We investigated the clinical-pathological characteristics and attempted to identify the predictive factors for kidney and patient survival in order to provide some clues for the pathogenesis and treatment of this rare entity.

## Materials and Methods

### Patients

**Figure 1:**
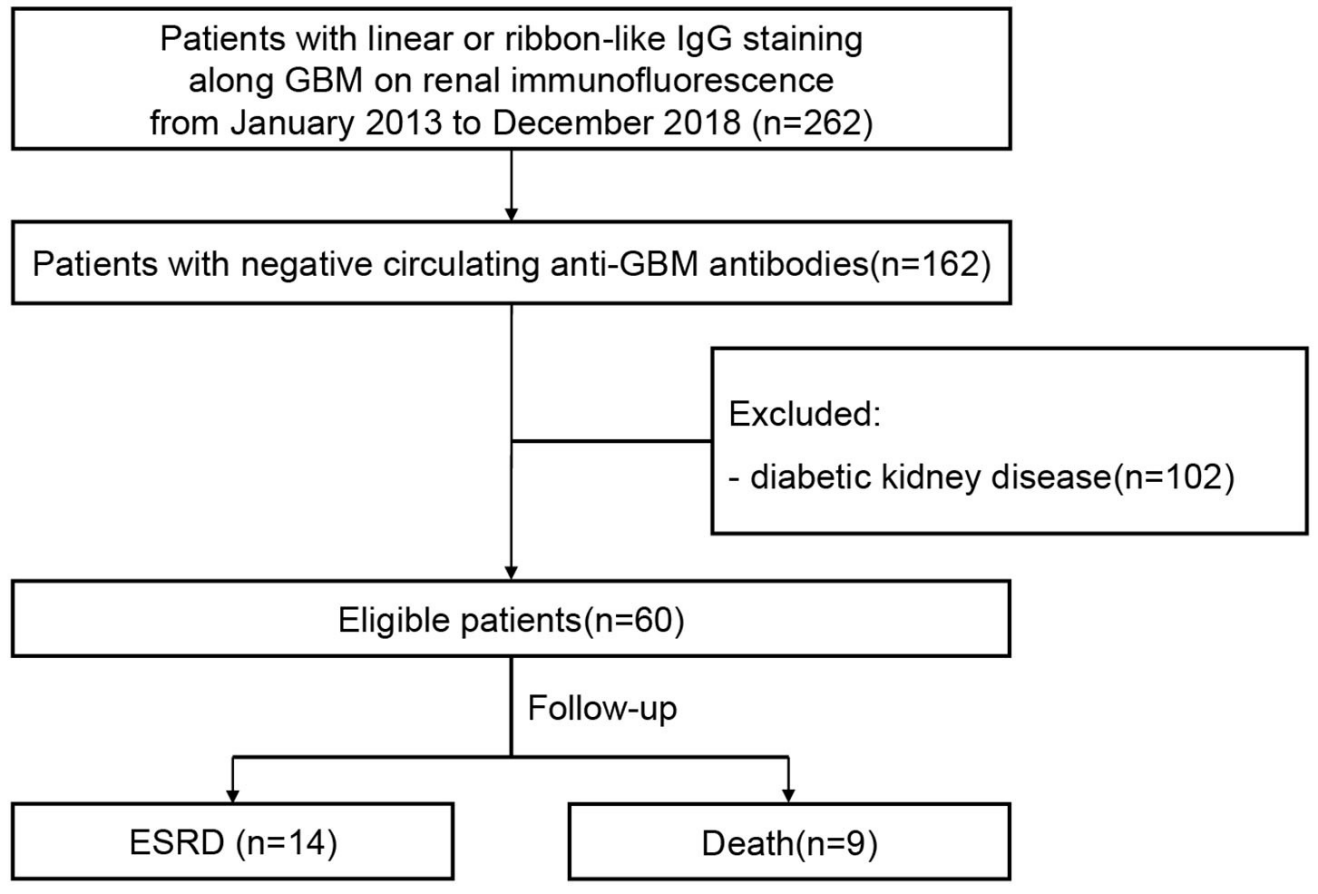
Flowchart of patient recruitment and follow-up.

Sixty patients with atypical anti-GBM disease identified at Peking University First Hospital were retrospectively analyzed from January 2013 to December 2018. The diagnostic criteria of “atypical anti-GBM disease” were defined as follows: 1. Immunofluorescence of renal specimens exhibited substantial linear deposit of IgG along GBM (staining intensity ≥1+); 2. Detection of circulating anti-GBM antibodies were negative examined by commercial ELISA kits (Euroimmun, Luebeck, Germany); 3. Patients with diabetic kidney disease were excluded. A study flow diagram is drawn to summarize the study procedure (Figure 1).

Demographic, clinical, and laboratory data were collected at the time of kidney biopsy and during follow-up. Renal insufficiency was defined as the serum creatinine >133 μmol/L on diagnosis. All patients were followed up until they met the endpoints. The primary endpoint (renal survival) was set as end-stage renal disease (ESRD) defined as dialysis dependence for >3 months. Patients who had not progressed to ESRD before death were treated as censored data when analyzing renal survival.

This study complied with the Declaration of Helsinki and was approved by the Ethics Committee of Peking University First Hospital.

### Kidney Pathology

Kidney biopsy was performed in all the 60 patients. The staining of IgG, IgA, IgM, C3, C1q, fibrinogen-fibrin related antigen (FRA), albumin, IgG subclasses and light chains were performed on frozen renal sections using fluorescein-conjugated rabbit/mouse anti-human IgG, IgA, IgM, C3c, C1q, FRA, albumin, light chain, IgG subclasses antibodies (Dako, Santa Clara, CA), and were evaluated under a fluorescence microscope (Nikon, Tokyo, Japan). The grades of staining intensity were ranged from 0+ to 4+. Light microscopy and electron microscopy examinations were performed as previously showed (20). All the pathological evaluations were performed by two renal pathologists blinded to each other.

### Statistical Analysis

SPSS statistical software (version22.0, IBM) was applied for statistical analysis. Quantitative data were presented as mean ± SD when complying with normal distribution, or as median (1/4, 3/4) when disobeying normal distribution. Qualitative data were presented as number (%). Comparison between continuous variables was conducted by *t*-test for normally distributing data or non-parametric test for non-normally distributing data. Differences between qualitative data were analyzed using χ^2^ or Fisher exact test. Univariate survival analysis was operated using both Kaplan-Meier analysis (log-rank test) and univariate COX regression analysis to explore potential prognostic predictors. Candidate variables were then enrolled together in a COX regression models to undergo multivariate survival analysis. Output results were exhibited as hazard ratios (HRs) along with 95% confidence intervals (95% CIs). The difference was considered statistically significant as *P*-value < 0.05.

## Results

### The Demographic and Clinical Features of Atypical Anti-GBM Patients

A total of 60 consecutive patients were retrospectively analyzed in this study, fitting the criteria of “atypical anti-GBM disease” from 2013 to 2018 (Table 1). They had a male predominance, and the ratio of male to female was 2.3:1. The ages of patients ranged from 19 to 87 years old, with an average age of 51.7 ± 15.6 years. 53.3% of patients were current or remote smokers. 13.3% of patients displayed prodromal infections before disease onset. 5.0% of patients manifested hemoptysis.

**Table 1:** Demographic and clinical characteristics of patients with atypical anti-GBM disease.

**Characteristic**	**Total patients (*N* = 60)**
**Demography**	
Male/female	42/18 (2.3/1)
Age, year	51.7 ± 15.6
**Clinical feature**	
Interval from onset to diagnosis, month	2.1 (1.1, 6.9)
Smoking, *n* (%)	32 (53.3)
Prodromal infection, *n* (%)	8 (13.3)
Hemoptysis, *n* (%)	3 (5.0)
^a^AKD and ^b^AKI, *n* (%)	27 (45.0)
Oliguria/anuria, *n* (%)	5 (8.3)
Hematuria, *n* (%)	38 (63.3)
Macroscopic hematuria, *n* (%)	4 (6.7)
Proteinuria, *n* (%)	56 (93.3)
24 h Proteinuria, g/24 h	2.7 (0.8, 6.3)
Nephrotic level proteinuria, *n* (%)	26 (43.3)
Nephrotic syndrome, *n* (%)	19 (31.7)
Serum albumin, g/L	34.2 (23.8, 41.2)
Serum creatinine on diagnosis, μmol/L	142.5 (87.8, 257.5)
Renal insufficiency, *n* (%)	32 (53.3)
Hemoglobin, g/L	118.1 ± 26.3
Serum C3, g/L^*^	0.94 ± 0.26 (*n* = 52)
Serum C4, g/L^*^	0.25 ± 0.07 (*n* = 52)
^c^ESR, mm/h	36.5 (17.0, 71.3) (*n* = 52)
^d^ANCA, *n* (%)	7 (14.0) (*n* = 50)
^e^MPO-ANCA/^f^PR3-ANCA/both	6/1/0
**Treatment**	
^g^ACEIs/^h^ARBs, *n* (%)	26 (43.3)
Immunosuppressive therapy, *n* (%)	34 (56.7)
steroids, *n* (%)	32 (53.3)
cytotoxic drugs, *n* (%)	18 (30.0)
Plasmapheresis, *n* (%)	4 (6.7)
**Outcome**	
Follow-up duration, month	35.7 ± 21.4
Progression to ESRD, *n* (%)	14 (23.3)
Death, *n* (%)	9 (15.0)
1-year renal survival, *n* (%)	50 (83.3)
1-year patient survival, *n* (%)	57 (95.0)

a AKD, acute kidney disease;

b AKI, acute kidney injury;

c ESR, erythrocyte sedimentation rate;

d ANCA, anti-neutrophil cytoplasmic antibodies;

e MPO, Myeloperoxidase;

f PR3, proteinase 3;

g ACEIs, angiotensin converting enzyme inhibitors;

h *ARBs, angiotensin receptor blocker*.

* *Normal range of serum C3: 0.6–1.5 g/L, normal range of serum C4: 0.12–0.36 g/L*.

Thirty eight (63.3%) patients exhibited hematuria and 4 of them had macroscopic hematuria. Proteinuria existed in 56 (93.3%) patients and 26 of them reached nephrotic level. The median level of proteinuria was 2.7 (0.8, 6.3) g/24 h. Nineteen (31.7%) patients presented with nephrotic syndrome. 45.0% (27/60) of patients presented with acute kidney disease(AKD), among them 18.5% (5/27) underwent oliguria or anuria. The median level of serum creatinine at diagnosis was 142.5 (87.8, 257.5) μmol/L, and over half of the patients (32/60, 53.3%) showed renal insufficiency at presentation. The serum C3 level, available in 52 patients, was normal in 49 and low in three. The serum C4 level, available in 52 patients, was normal in 51 and low in one. Anti-neutrophil cytoplasmic antibodies(ANCA) were detectable in serum of 14.0% (7/50) of the patients, among whom six were MPO-ANCA positive and one was PR3-ANCA positive.

### Kidney Pathology

All patients exhibited visible linear deposit of IgG along GBM with the intensity grade ranging from 1+ to 4+. Linear deposit of IgG could be observed at GBM (60/60, 100.0%), tubular basement membrane (37/60, 61.7%) and/or Bowmans' capsule (5/60, 8.3%). In 41 patients who had IgG light chains examined, all have both kappa and lambda chains deposit. IgG1 was the predominant subclass (27/59, 45.8%), followed by IgG2 (21/59, 35.6%), IgG4 (11/59, 18.6%), and IgG3 (7/59, 11.9%). Coexistence of IgA and IgM were shown in 27/60 (45.0%) and 33/60 (55.0%) patients. Complement deposits including C3 and C1q were found in 39 (65.0%) and 10 (16.7%) patients, respectively (Table 2).

**Table 2:** Pathological characteristics of patients with atypical anti-GBM disease.

**Characteristic**	**Total patients (*N* = 60)**
**Immunofluorescence**	
IgG linear deposition, *n* (%)	60 (100.0)
Intensity (scale 0~4+)	1.0 (1.0, 1.5)
Location (GBM/^a^TBM/Bowman's capsules), *n* (%)	60/37/5 (100.0/61.7/8.3)
IgG subclass (*n* = 59)	
IgG1/IgG2/IgG3/IgG4, *n* (%)	27/21/7/11 (45.8/35.6/11.9/18.6)
IgA deposit, *n* (%)	27 (45.0)
IgM deposit, *n* (%)	33 (55.0)
C3 deposit, *n* (%)	39 (65.0)
C1q deposit, *n* (%)	10 (16.7)
^b^FRA deposit, *n* (%)	20 (33.3)
Albumin deposit, *n* (%)	38 (63.3)
**Light microscopy**	
Number of glomeruli	25.0 (19.3, 36.0)
Crescent formation, *n* (%)	25 (41.7)
Percentage of crescents, %	27.3 (0.0, 49.7)
^c^TA/IF, *n* (%)	58 (96.7)
**Electron microscopy**	
Electric dense deposit, *n* (%)	33 (55.9) (*n* = 59)
Combined ^d^GN, *n* (%)	35 (58.3)
^e^IgAN (including ^f^HSP-GN), *n* (%)	12 (20.0)
^g^MN, *n* (%)	8 (13.3)
^h^MPGN, *n* (%)	6 (10.0)
^i^AAV, *n* (%)	4 (6.7)
^j^FSGS, *n* (%)	3 (5.0)
^k^TBMN *n* (%)	1 (1.7)
^l^TMA, *n* (%)	1 (1.7)

a TBM, tubular basement membrane;

b FRA, fibrinogen-fibrin related antigens;

c TA/IF, tubular atrophy and interstitial fibrosis;

d GN, glomerulonephritis;

e IgAN, IgA nephropathy;

f HSP-GN, Henoch–Schönlein purpura glomerulonephritis;

g MN, membranous nephropathy;

h MPGN, membranoproliferative glomerulonephritis;

i AAV, ANCA-associated vasculitis;

j FSGS, focal segmental glomerulosclerosis;

k TBMN, thin basement membrane nephropathy;

l *TMA, thrombotic microangiopathy*.

41.7% (25/60) of all cases were observed of crescent formation and the percentage of crescent was (34.7 ± 23.5)% in those patients. Five of them had crescentic glomerulonephritis (defined by diffuse crescents occupying >50% of the glomeruli). In patients with crescents, the average proportion of cellular, cellulofibrous, and fibrous crescents was 25.8, 52.8, and 21.4%, respectively. 56.0% (14/25) of those patients showed crescents in synchrony, the remaining showed a mixture of acute and chronic lesions. There was a positive correlation between the percentage of crescents and the serum creatinine at diagnosis (*r* = 0.427, *P* = 0.001). Almost all patients showed tubular atrophy and interstitial fibrosis (58/60, 96.7%), interstitial inflammatory cells infiltration (55/60, 91.7%) and arteriole injury (59/60, 98.3%). Electric dense deposit was observed in 33/59 (55.9%) patients. Foot process effacement of podocyte appeared in most of the patients (55/59, 93.2%). 58.3% (35/60) of all patients combined with other glomerulonephritis, including IgA nephropathy (12/60, 20.0%), membranous nephropathy (8/60, 13.3%), membranoproliferative glomerulonephritis (6/60, 10.0%), ANCA-associated vasculitis (4/60, 6.7%), focal segmental glomerulosclerosis (3/60, 5.0%), thin basement membrane nephropathy (1/60, 1.7%), thrombotic microangiopathy (1/60, 1.7%) (Table 2).

### Treatment and Outcome

26/60 (43.3%) patients received only angiotensin converting enzyme inhibitors (ACEIs) or angiotensin receptor blocker (ARBs). Immunosuppressive therapies that were defined as administration of steroids and/or cytotoxic drugs, were applied in 34/60 (56.7%) patients. Among them, 16 patients received administration of steroids combined with cytotoxic drugs, 16 patients received steroids alone and two patients received cytotoxic drugs alone. In 18 patients who received cytotoxic drugs, 14 of them were treated with cyclophosphamide (CTX), the remaining with cyclosporine (two patients), tacrolimus (one patient) and mycophenolate mofetil (one patient), respectively. Plasma exchange was performed in 4/60 (6.7%) patients (Table 1).

**Figure 2:**
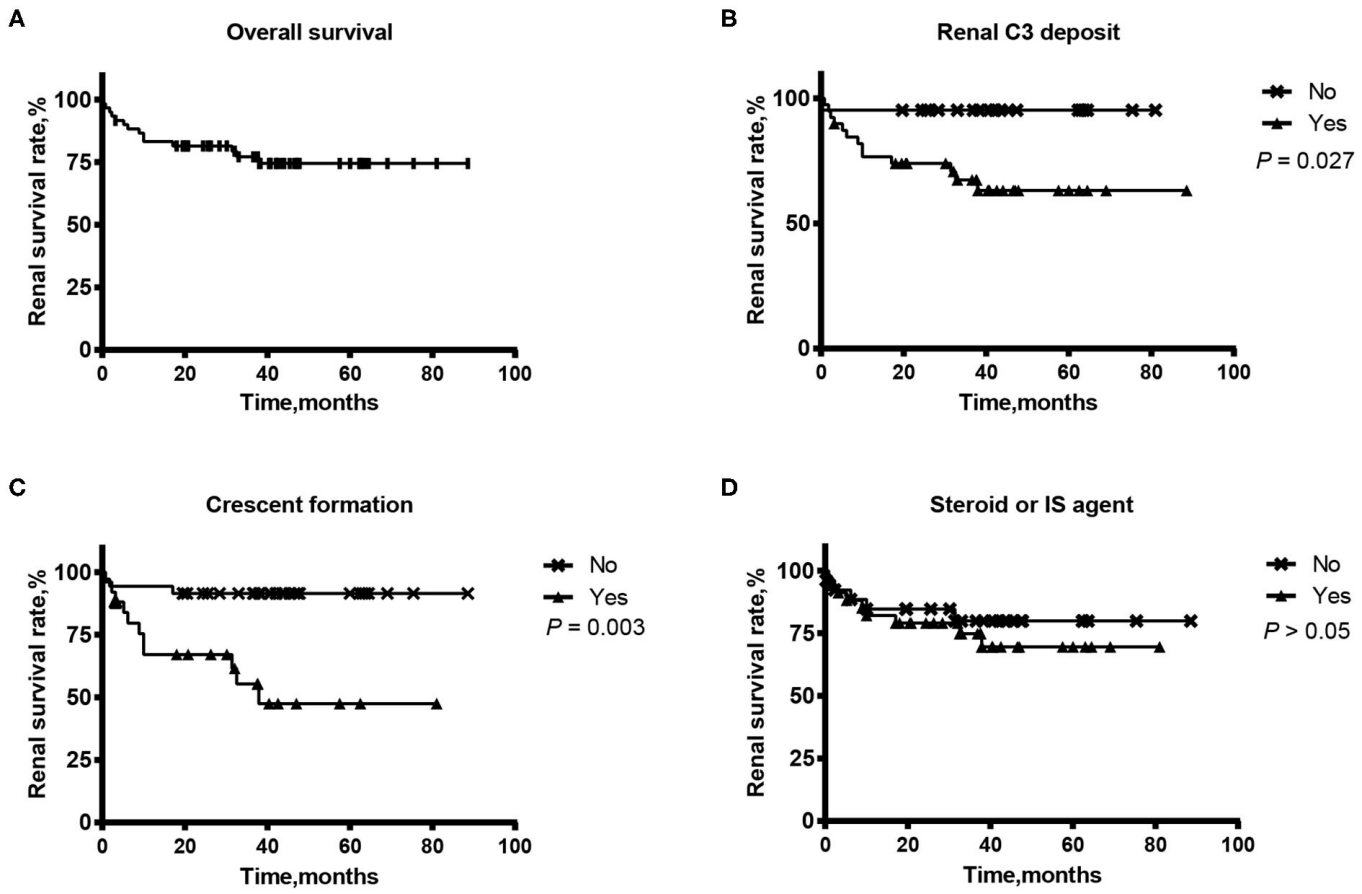
Renal survival of a Chinese cohort of 60 patients with atypical anti-GBM disease: Overall renal survival **(A)** and according to the renal deposit of C3 **(B)**, the crescent formation in glomeruli **(C)** and the administration of steroids or immunosuppressant (IS) agents **(D)**.

The follow-up ranged from 3 months to 89 months with an average of 35.7 ± 21.4 months. During follow-up, 14/60 (23.3%) patients progressed to ESRD. The 1-year renal survival rate was 83.3% (50/60). The prognostic values of clinical-pathological parameters and therapeutic strategies for kidney outcome were evaluated using Kaplan-Meier analysis (log-rank test) and Cox regression analysis, shown in Figure 2, Table 3. After univariate survival analysis, we found that the level of serum creatinine on diagnosis, level of serum C3, intensity of kidney C3 staining, kidney C1q positive staining, percentage of crescents and plasmapheresis were potential risk factors for ESRD. Multivariate analysis showed that serum creatinine on diagnosis [per 200 μmol/L increase, HR (95% CI): 2.663 (1.372, 5.172), *P* = 0.004], serum C3 (per 0.1 g/L increase, HR (95% CI): 0.689 (0.483, 0.984), *P* = 0.040) and the intensity of kidney C3 staining (per 1+ increase, HR (95% CI): 2.770 (1.115, 6.877), *P* = 0.028) were independent predictive factors for kidney outcome. Immunosuppressant therapies had no significant association with kidney outcome.

**Table 3:** Potential prognostic factors for kidney outcome by univariate and multivariate COX regression analysis.

**Variable**	**Univariable analysis (*****N*** **=** **60)**		**^a^Multivariable analysis (*****N*** **=** **52)**	
	***P*-value**	**HR (95% CI)**	***P*-value**	**HR (95% CI)**
Gender (female)	0.528	0.663 (0.185, 2.379)	-	-
Age	0.151	1.026 (0.991, 1.063)	-	-
Hematuria (0 = none; 1 = microscopic; 2 = macroscopic)	0.090	2.227 (0.883, 5.622)	-	-
Proteinuria (0 = none; 1 = non-nephrotic; 2 = nephrotic)	0.327	1.566 (0.639, 3.839)	-	-
SCr on diagnosis (increased by 200 μmol/L)	***<0.001***	2.355 (1.598, 3.471)	***0.004***	2.663 (1.372, 5.172)
Serum C3 (increased by 0.1g/L) (*n* = 52)	***0.002***	0.608 (0.445, 0.830)	***0.040***	0.689 (0.483, 0.984)
Kidney IgG staining >1+	0.271	1.803 (0.631, 5.154)	-	-
IgG deposit on TBM and/or Bowman's capsule	0.272	0.555 (0.195, 1.585)	-	-
Kidney C3 staining intensity (increased by 1+)	***0.006***	2.170 (1.252, 3.762)	***0.028***	2.770 (1.115, 6.877)
Kidney C1q positive staining	***0.041***	3.126 (1.045, 9.353)	0.780	0.805 (0.175, 3.699)
Percentage of crescents (increased by 10%)	***0.009***	1.258 (1.059, 1.494)	0.775	0.940 (0.616, 1.435)
Combined with other GN	0.364	1.711 (0.536, 5.459)	-	-
ACEIs/ARBs	0.484	0.677 (0.227, 2.021)	-	-
Steroids	0.340	1.703 (0.570, 5.087)	-	-
Cytotoxic drugs	0.597	1.343 (0.450, 4.010)	-	-
Plasmapheresis	***0.019***	4.692 (1.295, 17.005)	0.849	0.749 (0.038, 14.796)

a *Multivariable analysis was performed in a subgroup of 52 patients, of which the values of serum C3 were available at the presentation. Bold values represent P < 0.05*.

Nine (15.0%) patients died during follow-up. The 1-year patient survival rate was 95.0% (57/60). Four patients died of severe pneumonia and respiratory failure. One died of acute myocardial infarction. Four died of unknown reasons. In the nine died patients, four were dialysis dependence lasting for more than 3 months before death and were regarded as meeting the primary endpoint. The remaining five patients who did not progressed to ESRD before death were treated as censored data when analyzing renal survival. The predictive indicators for death were evaluated using Kaplan-Meier analysis (log-rank test) and Cox regression analysis, shown in Table 4. After univariate survival analysis, we found that age, the intensity of kidney C3 staining, and the percentage of crescents were potential risk factors for death. However, multivariate analysis did not come out with any independent predictive factors for death.

**Table 4:** Potential prognostic factors for patient outcome by univariate and multivariate COX regression analysis.

**Variable**	**Univariable analysis (*****N*** **=** **60)**		**Multivariable analysis (*****N*** **=** **60)**	
	***P*-value**	**HR (95% CI)**	***P*-value**	**HR (95% CI)**
Gender (female)	0.461	0.552 (0.113, 2.684)	-	-
Age	***0.028***	1.054 (1.006, 1.105)	0.080	1.046 (0.995, 1.099)
Hematuria (0 = none; 1 = microscopic; 2 = macroscopic)	0.634	1.291 (0.451, 3.695)	-	-
Proteinuria (0 = none; 1 = non-nephrotic; 2 = nephrotic)	0.773	0.850 (0.281, 2.568)	-	-
SCr on diagnosis (increased by 200 μmol/L)	0.457	1.207 (0.735, 1.981)	**-**	-
Serum C3 (increased by 0.1 g/L) (*n* = 52)	0.057	2.010 (0.980, 4.120)	***-***	-
Kidney IgG staining >1+	0.894	1.101 (0.267, 4.533)	-	-
IgG deposit on TBM and/or Bowman's capsule	0.377	2.032 (0.422, 9.797)	-	-
Kidney C3 staining intensity (increased by 1+)	***0.012***	1.937 (1.155, 3.248)	0.263	1.664 (0.682, 4.060)
Kidney C1q positive staining	0.710	1.347 (0.279, 6.500)	-	-
Percentage of crescents (increased by 10%)	***0.016***	1.272 (1.045, 1.547)	0.173	1.156 (0.938, 1.425)
Combined with other GN	0.755	0.811 (0.217, 3.030)	-	-
ACEIs/ARBs	0.191	0.351 (0.073, 1.688)	-	-
Steroids	0.140	3.269 (0.679, 15.741)	-	-
Cytotoxic drugs	0.812	1.183 (0.296, 4.735)	-	-
Plasmapheresis	0.628	1.673 (0.209, 13.396)	-	-

*Bold values represent P < 0.05*.

### Comparison Between Atypical Anti-GBM Patients With and Without Crescent Formation

41.7% patients of the whole cohort presented with crescent formation in renal histological examinations. The clinical and pathological features of patients with and without crescent formation were compared (Table 5). The patients with crescents presented with more significant male predominance (84.0 vs. 60.0%, *P* = 0.046), higher levels of SCr at diagnosis **[**206.8 (123.9, 372.7) μmol/L vs. 109.9 (82.7, 161.5) μmol/L, *P* = 0.003], higher frequency of kidney C3/IgA/IgM deposit (92.0 vs. 45.7%, *P* < 0.001; 64.0 vs. 31.4%, *P* = 0.012; 72.0 vs. 42.9%, *P* = 0.025), worse kidney survival (ESRD, 44.0 vs. 8.6%, *P* = 0.001) and higher proportion of death (28.0 vs. 5.7%, *P* = 0.044). More patients received immunosuppressive therapy in the group with crescent formation (76.0 vs. 42.9%, *P* = 0.011). Besides, hemoptysis (three cases) was only found in the patients with crescents.

**Table 5:** Comparison of clinical and pathological features between patients with and without crescents.

**Characteristic**	**With CGN^a^**	**Without CGN**	***P-*value**
	**(*n* = 25)**	**(*n* = 35)**	
**Clinical feature**			
Male/female	21/4	21/14	***0.046***
Age, year	52.4 ± 16.8	51.2 ± 14.9	0.784
Smoking, *n* (%)	18 (72.0)	14 (40.0)	***0.014***
Prodromal infection, *n* (%)	7 (28.0)	1 (2.9)	***0.015***
Hemoptysis, *n* (%)	3 (12.0)	0 (0.0)	0.133
AKD or AKI, *n* (%)	14 (56.0)	13 (37.1)	0.148
Oliguria/anuria, *n* (%)	2 (8.0)	3 (8.6)	1.000
Hematuria, *n* (%)	21 (84.0)	17 (48.6)	***0.005***
24 h Proteinuria, g/24 h	3.8 (2.0, 6.9)	1.5 (0.5, 6.3)	0.089
Serum albumin, g/L	31.4 (23.9, 36.5)	36.2 (23.2, 42.8)	0.195
SCr on diagnosis, μmol/L	206.8 (123.9, 372.7)	109.9 (82.7, 161.5)	***0.003***
ANCA, n/N (%)	6/24 (25.0)	1/26 (3.8)	0.081
**Pathology**			
IgG deposit intensity (scale 0~4+)	1.0 (1.0, 2.3)	1.0 (1.0, 1.5)	0.188
IgA deposit, *n* (%)	16 (64.0)	11 (31.4)	***0.012***
IgM deposit, *n* (%)	18 (72.0)	15 (42.9)	***0.025***
C3 deposit, *n* (%)	23 (92.0)	16 (45.7)	***<0.001***
C1q deposit, *n* (%)	6 (24.0)	4 (11.4)	0.349
FRA deposit, *n* (%)	12 (48.0)	8 (22.9)	***0.042***
Electric dense deposit, n/N (%)	19/24 (79.2)	14/35 (40.0)	***0.003***
**Treatment**			
ACEIs/ARBs, *n* (%)	6 (24.0%)	20 (57.1)	***0.011***
Immunosuppressive therapy, *n* (%)	19 (76.0)	15 (42.9)	***0.011***
Steroids, *n* (%)	19 (76.0)	13 (37.1)	***0.003***
Cytotoxic drugs, *n* (%)	11 (44.0)	7 (20.0)	***0.046***
Plasmapheresis, *n* (%)	4 (16.0)	0 (0.0)	0.054
**Outcome**			
Follow-up duration, month	27.4 ± 21.1	41.9 ± 19.7	***0.009***
Progression to ESRD, *n* (%)	11 (44.0)	3 (8.6)	***0.001***
Death, *n* (%)	7 (28.0)	2 (5.7)	***0.044***
1-year renal survival, *n* (%)	17 (68.0)	33 (94.3)	***0.012***
1-year patient survival, *n* (%)	22 (88.0)	35 (100.0)	0.067

a *CGN: crescentic glomerulonephritis. Bold values represent P < 0.05*.

## Discussion

To our best knowledge, the present study comprised the largest cohort of atypical anti-GBM disease. Atypical anti-GBM disease manifested milder clinical features and better kidney outcomes compared to classical anti-GBM disease. Though rather heterogeneous, a substantial number of the patients had complement activation and crescent formation. Patients having crescents presented with more severe clinical course and worse renal and patient outcomes than those without crescents. It is of note that nearly a quarter of these patients progressed to ESRD and 9/60 patients died with a median follow up of 36 months. The poor kidney and patient prognosis, not favorable as expected, emphasizes the attention to atypical anti-GBM disease from physicians. Our study showed that the immunosuppressive intervention was not associated with kidney or patient outcome. In future, prospective and controlled studies might be needed to address the optimal therapeutic regimen.

Our retrospective study unearthed that the clinical and pathological features of patients with atypical anti-GBM disease were rather heterogeneous, and milder than classical anti-GBM patients. Less than half of the patients underwent a course of AKD or AKI. Kidney injuries were much slighter than that in classical anti-GBM disease, manifested as less crescent formation and lower levels of SCr at presentation (21, 22). However, the degree of kidney impairment varied as 1/5 of patients exhibited SCr levels >300 μmol/L, while 1/2 of patients presented normal kidney function. Although half of the patients were current or former smoker, the manifestation of hemoptysis was rather rare in these patients, in contrast to ~40–60% of classical anti-GBM patients presenting pulmonary involvement (11). Distinguished from mild to moderate proteinuria in classical anti-GBM disease (7), the degree of proteinuria was much more severe in atypical patients. Nearly half of the patients showed nephrotic-range proteinuria and 1/3 of them suffered from nephrotic syndrome. Almost all patients with atypical anti-GBM disease showed tubular-interstitial and arteriole injury, which was less common in typical anti-GBM disease. These histopathological features implied a more chronic course in atypical anti-GBM disease.

In our cohort of atypical patients, around half of all cases had crescent formation. Though less than classical patients (21), the percentage of crescents were associated with serum creatinine on diagnosis. A further comparison analysis showed that the kidney outcomes of patients with crescents were worse than those without crescents. Univariate survival analysis showed that the percentage of crescents was associated with renal survival. These results were similar to previous reports in classical anti-GBM disease that the proportion of crescents was an independent predictor for ESRD (23). It is of notice that nearly all patients with crescents had positive C3 staining, in contrast to merely half in patients without crescents. Moreover, higher level of serum C was an independent protective factor and the intensity of kidney C3 staining was an independent risk factor for kidney outcome in this cohort. Renal C3 deposit generally implies the activation of complement system in the kidneys, which promotes the formation of membrane attack complex to damage the tissues (24). As previously reported, almost all patients with anti-GBM disease have C3 deposit in glomeruli (21). Therefore, we speculated that the deposited linear IgG in a substantial atypical anti-GBM patients might also act as “classical pathogenic antibodies,” which causes the activation of complement resulting in kidney injuries and crescent formation. However, the positive rate of C3 staining is lower in patients with atypical anti-GBM disease, which again reflects the heterogeneity in these patients and summons further investigations on the renal complement activation and its association with kidney outcome.

Previous studies have proven that the combination of plasmapheresis, steroids and CTX could improve renal and patient outcomes in classic anti-GBM disease (25–27). However, there were no unified recommendations for the treatment of atypical anti-GBM disease at present, given the heterogeneity of these patients (15, 18, 28). Treatments varied in different patients, which were highly dependent on the clinical judgments by physicians. In our cohort, only half of the patients received immunosuppressive treatments and 1/10 received plasmapheresis. Patients received steroids or immunosuppressant agents were usually those presented with more severe renal damage. A higher proportion of patients receiving steroids or immunosuppressant agents presented with crescent formation and renal dysfunction. The heterogeneity of treatments made it difficult to investigate on the association of immunosuppressive therapy and renal outcome in the current study. Considering a relatively high incidence of complement activation and poor renal outcome, the role of immunosuppressive treatment in atypical anti-GBM disease may need to be further explored in future studies.

Concurrent AAV and MN in patients with anti-GBM disease had been well-documented in previous articles (2), and sporadically IgAN (29, 30), HSP-GN (31), TMBN (8), TMA (32) et al. In our present study, a high proportion (58.3%) of patients with atypical anti-GBM disease coexisted with other glomerular diseases including IgAN, MN, MPGN, FSGS, TMBN, AAV, and TMA. Typically, those diseases presented no linear IgG deposit alongside GBM, therefore, we speculated that they might have underlying relations with the occurrence of anti-GBM disease in this rare entity. The local glomerular damage caused by the already existing glomerular diseases exposed the sequestered autoantigens in GBM and then elicited autoimmune responses toward the GBM. Another explanation might be that the anti-GBM antibodies elicited immuno-inflammatory reactions in glomeruli, caused local tissue injury and facilitated other glomerular diseases.

There were several underlying explanations for the absence of circulating anti-GBM antibodies: (1) Instead of α3(IV)NC1, antibodies of some patients recognized unconventional antigens located on GBM which were beyond routine assays. Serum anti-GBM antibodies could be detected by indirect immunofluorescence using normal kidney tissues in a few patients of our cohort, which collaborated this hypothesis (data not shown). (2) Antibodies with low affinity could only be discovered by higher sensitive assays such as western blot and biosensor experiments rather than routine methods (33). (3) Similar like other autoimmune diseases, during the reconstruction of immune homeostasis in disease retrieval, the production of antibodies paused and circulating antibodies were obliterated by liver, but the tissue antibodies were hard to eliminate and presented a longer half life time (34).

There are several limitations of this study. First of all, the follow-up duration is short for survival analysis for this rare disease. Secondly, the treatments of patients had a high heterogeneity in our cohort, thus the role of immunosuppressive therapy in patient and kidney survival might be underestimated due to data bias. Lastly, the current study comprised of patients from Chinese population which might be lack of generalizability to other races.

## Data Availability Statement

The raw data supporting the conclusions of this article will be made available by the authors, without undue reservation.

## Ethics Statement

The studies involving human participants were reviewed and approved by the Ethics Committee of Peking University First Hospital. The patients/participants provided their written informed consent to participate in this study.

## Author Contributions

CS participated in the research design, performance of the research, data analysis, and article writing. XJ participated in the research design, data analysis, and article writing. ZC participated in the research design and data analysis. XY participated in the performance of the research and data analysis. MZ participated in the research design and article modification. All authors were involved in revising the manuscript and approved the final version.

## Conflict of Interest

The authors declare that the research was conducted in the absence of any commercial or financial relationships that could be construed as a potential conflict of interest. The handling editor declared a past co-authorship with several of the authors XJ, ZC, and MZ.
